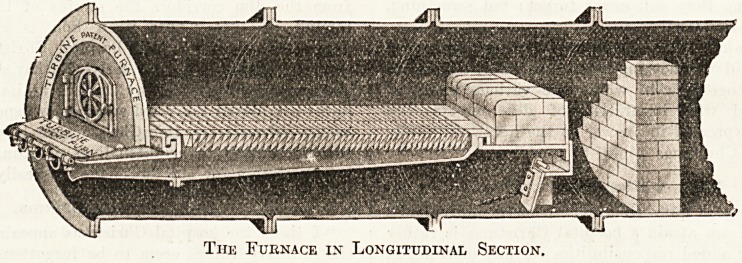# An Invention to Improve Both Efficiency and Economy

**Published:** 1920-12-25

**Authors:** 


### THE TURBINE FURNACE.

### An Invention to Improve both Efficiency and Economy.

THE steam boiler has long been a necessary part of the equipment of all hospitals of any size. Steam offers the most convenient method of transporting heat to any part of the building where it may be required. It can be employed directly in radiators to heat the wards, the operating-theatre, and other parts of the building; or it may be employed to heat the water that is used in a hot-water system of radiators; it is available for heating water for general purposes all over the hospital, also for sterilisers, for cooking, and numerous other purposes.

### ECONOMY.

At the present time, economy, wherever it can be obtained is the order of the day; and the turbine furnace has been designed to secure economy in the burning of the coal that is used to generate steam in the steam boilers. In the Lancashire boiler, which is so largely used in hospitals, and which the Americans call a "tank" boiler, there is a long cylinder, of varying dimensions, according to the quantity of steam required, from 20 feet to 30 feet long, and from 5 feet to 9 feet in diameter; and it is pierced by two smaller cylinders, running the full length of the containing cylinder; they are called the flue-tubes, and the furnaces where the coal is burnt are fixed at the front end of both the tubes. When the boiler is at work there is enough water in it to completely cover the flue-tubes, and to have a certain depth of water above them, the remainder of the space holding the steam that is being generated from the water. In the ordinary Lancashire boiler furnace, fire-bars, somewhat similar, but very much larger than those of a domestic fire-grate, are placed across the flue-tubes, a number of them being fixed one behind the other, with small spaces between them for the air to pass upwards and the ashes to pass downwards; the spaces in the flue-tubes, below the fire-bars, form the ash-pits. It is well known that economical consumption of the coal is very rarely attained with the ordinary furnace grate; it depends very largely upon the stoker, and the human element is generally very much in evidence.

As the writer understands the matter, the Turbine Furnace is designed to enable cheap, low grades of fuel to be burnt in the hand-fired furnace with economy, and to raise the efficiency of the boiler furnace under all conditions, whatever kind of fuel is burnt. There is room for considerable improvements in this matter; the ordinary boiler furnace and the ordinary arrangement of the boiler rarely gives more useful heat in the steam than 50 to 55 per cent. of that liberated by the coal burnt on the fire-bars.

### DESCRIPTION OF THE FURNACE.

In the production of the Turbine Furnace Co. of London, to whom we are indebted for a number of descriptive details, the fire-bars are divided into sections longitudinally; each section consists of a number of narrower fire-bars occupying one-fourth, one-fifth, or one-sixth of the width of the flue-tubes in place of the whole width. Each section is held in a trough, which in its turn is held between the front of the flue-tube—the "dead plate," as it is called—and the bridge at the back of the furnace. The fire-bars themselves are constructed so that the spaces between them are curved in section, something on the lines of the turbine blades of an impulse turbine, hence the name. The fire-bars are locked together when in position by what is practically a dovetail between successive bars, and the contour of the front and back of each bar are such as to form the curved spaces referred to. The lower parts of adjacent bars are inclined to each other at an angle of 45 degrees, and the upper parts at 60 degrees, so that the air which passes up through the curved spaces expands in the upper portion. The troughs diminish in sectional area from the front of the furnace to the back.

THE FURNACE IN LONGITUDINAL SECTION.

### MANY ADVANTAGES.

At the front end of each trough a trumpet-shaped tube is fixed; it is formed of the frustums of two cones, the necks of the cones being together near the front of the flue-tubes. At the entrance of the outer cone is a small steam nozzle, and the passage of the steam through the nozzle into the cone beyond draws air in through the outer cone and forces it along the trough and up through the spaces between the fire-bars. This is a modification of a well-known form of "forced draught," but the consumption of steam by the nozzles is very much smaller than with the older form of that kind of draught apparatus. A longitudinal

**The Turbine Furnace—(*continued*).**

section of the furnace as fixed in a Lancashire boiler is shown in the drawing, from which the parts that have been described will be seen. It will be noted that the ashes do not go down into the lower part of the flue-tube. Very little ash is made, and it is easily cleaned out by the boiler-man by the aid of a special tool which the makers provide. In addition to the above, there is a fire-brick arch built in the flue-tube about 18 in. farther inwards than the fire-brick bridge that usually terminates the boiler furnace, and an arrangement is made, by means of a door that can be opened from the front of the boiler, to admit air to the space between the two fire-brick erections. Uneconomical burning of fuel is often due either to the carbon of the coal being only oxidised to carbonic oxide instead of carbonic acid or to the carbonic acid that has been formed in one part of the furnace taking up another atom of carbon to each molecule of carbonic acid and forming two molecules of carbonic oxide. In either case there is a loss of about two-thirds of the heat units that ought to be provided by the combustion of the coal. The chamber between the fire-brick extensions provides for secondary combustion of the carbonic oxide, thereby increasing the efficiency of the furnace. There is a cleaning-door at the same place where the air-door is fixed, and the flue-dust that is a prominent feature in all Lancashire boilers can be easily removed through the cleaning-door.

Other advantages of the turbine furnace are that it can easily and quickly be fixed to any boiler—Lancashire, Cornish, water-tube, fire-tube, or any other. Careful tests have been made of the working of the furnace against the ordinary boiler furnace, and the results obtained are eminently satisfactory.

## Figures and Tables

**Figure f1:**